# Progesterone and Related Compounds in Hepatocellular Carcinoma: Basic and Clinical Aspects

**DOI:** 10.1155/2013/290575

**Published:** 2013-01-16

**Authors:** Yao-Tsung Yeh, Chien-Wei Chang, Ren-Jie Wei, Shen-Nien Wang

**Affiliations:** ^1^Department of Medical Laboratory Sciences and Biotechnology, Fooyin University, Kaohsiung 83102, Taiwan; ^2^Cancer Center and Division of General & Gastroenterological Surgery, Department of Surgery, Kaohsiung Medical University Hospital, Kaohsiung 80756, Taiwan; ^3^Department of Pathology, Kaohsiung Armed Forces General Hospital, Kaohsiung 80284, Taiwan; ^4^Department of Surgery, Faculty of Medicine, College of Medicine, Kaohsiung Medical University, Kaohsiung 80756, Taiwan; ^5^Division of Hepato-Pancreatico-Biliary Surgery, Kaohsiung Medical University Hospital, Kaohsiung 80756, Taiwan

## Abstract

Primary liver cancer is the fifth most common cancer worldwide and the third most common cause of cancer mortality. Hepatocellular carcinoma (HCC) accounts for 85% to 90% of primary liver cancers. Major risk factors for HCC include infection with HBV or HCV, alcoholic liver disease, and most probably nonalcoholic fatty liver disease. In general, men are two to four times more often associated with HCC than women. It can be suggested that sex hormones including progesterone may play some roles in HCC. Rather, very limited information discusses its potential involvement in HCC. This paper thus collects some recent studies of the potential involvement of progesterone and related compounds in HCC from basic and clinical aspects. In addition, two synthetic progestins, megestrol acetate (MA) and medroxyprogesterone acetate (MPA), will be discussed thoroughly. It is noted that progesterone can also serve as the precursor for androgens and estrogens produced by the gonadal and adrenal cortical tissues, while men have a higher incidence of HCC than women might be due to the stimulatory effects of androgen and the protective effects of estrogen. Eventually, this paper suggests a new insight on the associations of progesterone and related compounds with HCC development and treatment.

## 1. Introduction

Primary liver cancer is the fifth most common cancer worldwide and the third most common cause of cancer mortality [1]. Hepatocellular carcinoma (HCC) accounts for 85% to 90% of primary liver cancers. HCC has several interesting epidemiologic features including dynamic temporal trends; marked variations among geographic regions, racial and ethnic groups, and between men and women; and the presence of several well-documented environmental potentially preventable risk factors. Most HCC cases (80%) occur in either sub-Saharan Africa or in Eastern Asia. China alone accounts for more than 50% of the world's cases. Other high-rate areas include Senegal, Gambia, and South Korea [2].

Major risk factors for HCC include infection with hepatitis B virus (HBV) or hepatitis C virus (HCV), alcoholic liver disease, and most probably nonalcoholic fatty liver disease [3]. In general, men are two to four times more often associated with HCC than women. Epidemiological reports indicate that, regardless of etiologies, the incidence of HCC is higher in males than in females with the male : female ratio usually averaging between 2 : 1 and 4 : 1 [2]. The ratio of men to women is more pronounced in areas with a high HCC incidence [4]. A part of this increased risk among men is explained by their higher frequency of viral hepatitis and alcoholic cirrhosis. A statistical analysis indicated that age at menopause is an important and significant predictor, increasing HCC risk 24% for each later year of menopause (odd ratio = 1.24, *P* < 0.001) [5], implicating that female sex hormones may be associated with HCC risk or development. However, the reason(s) for this residual difference in HCC risk between men and women is unknown and might be related to the carcinogenic effect of testosterone [6]. In a rat model, testosterone appears to be a growth factor for Morris hepatoma 7787 [7]. Additionally, epidemiologic and animal studies have suggested that men have a higher incidence of HCC than women which might be due to the stimulatory effects of androgen and the protective effects of estrogen [8]. Substituted androgens have been associated with the development of HCC in patients with Fanconi's anemia [9] and aplastic anemia [10]. These findings suggest that androgens may be implicated in the etiology of HCC [11]. In an animal experiment, exogenous and endogenous estradiol/active estrogen can suppress chemical-agent induced hepatocarcinogenesis in a rat, suggesting that estrogen receptors (ERs) may be involved in the inhibition of malignant transformation of preneoplastic liver cells [11]. Pregnancy, which increases serum estrogen levels about 100-fold, was found to exert a protective effect against HCC, and the protection increased with the number of FTP (full-term pregnancies) [12].

Based on available clinical information, chronic hepatitis C appears to progress more rapidly in men than in women, and cirrhosis is predominately a disease of men and postmenopausal women [13]. A larger number of women with advanced fibrosis (cirrhosis) were identified among menopausal women in chronic hepatitis C virus infection [14]. Cirrhosis frequently associates with HCC and hence can be considered a premalignant condition. Indeed, the majority of patients worldwide with HCC have underlying cirrhosis [15]. Both HBV and HCV promote cirrhosis, which is found in 80%–90% of patients with HCC. The 5-year cumulative risk of developing HCC for patients with cirrhosis ranges between 5% and 30%, depending on etiology, region or ethnicity, and stage of cirrhosis [16]. Interestingly, cirrhotic patients with HCC have significantly lower plasma concentrations of testosterone, dihydrotestosterone, and dehydroepiandrosterone than patients with cirrhosis alone [17]. Low levels of testosterone in male HCC patients and high levels of progesterone in cirrhosis patients have been observed [18]. It is controversial that high levels of progesterone are associated with premalignant cirrhosis. Do the higher progesterone levels contribute to HCC development? It is noted that the HCC risk was inversely related to the age at natural menopause. Oophorectomy performed at age 50 or younger during premenopausal years was also a risk factor for HCC [12], suggesting that at least female sex hormones including progesterone or estrogen may be protective against HCC.

## 2. Potential Involvement of Sex Hormones in Hepatocellular Carcinoma

Sex hormones such as estrogens, progestins, and androgens are hydrophobic ligands, which bind to transcription factors belonging to the superfamily of intracellular receptors. These receptors can be activated by the cognate ligand or in its absence, by posttranslational modifications elicited through the intracellular signaling of membrane receptors, also called nongenomic actions [19, 20]. Upon ligand binding, receptor activation occurs via diversified pathways involving genomic or nongenomic actions [21]; that is, the activated receptor may directly bind to the DNA-responsive elements in the regulatory regions of these genes (genomic actions) or may influence other pathways involved in cell proliferation by interfering with specific proteins in the cytoplasm or in the nucleus (non-genomic actions). Regarding the actions of sex hormones in HCC, their corresponding receptors should be always considered.

A novel cancer phenotype in which mice lacking hepatic androgen receptor (AR) developed more undifferentiated tumors and larger tumor size at the metastatic stage, which AR could orchestrate intrahepatic signaling hierarchies and cellular behaviors, consequently affect HCC progression [22]. Rather, higher androgen levels are frequently associated with HCC development. On the other hand, the incidence of ER content is highly variable according to the different authors, but study groups are not large enough. For the largest study group containing 66 HCC cases, ER content was found in 26 cases [23]. Rather, the presence or absence of progesterone receptor (PR), ER, and AR in HCC and their titers did not have any correlation with alcohol abuse, serum alpha-fetoprotein (AFP) levels, hepatitis B virus markers, or histopathologic types of the tumor [24].

Tamoxifen, a selective estrogen receptor modulator, is one of the most hormonal therapies used in breast cancer that can induce cell apoptosis through protein kinase C, MAPK, c-Myc, and so forth [25]. Interestingly, tamoxifen could also induce apoptosis of HepG2 cells in a dose-dependent fashion and reduced survivin transcript and mTOR activity of these cells [26]. A clinical study used tamoxifen to treat patients with expression of wild-type ER in HCC that has revealed a benefit to reduce tumor size [27] (Table 1). Furthermore, tamoxifen can also independently act without expression of ER in HCC [28]. However, a clinical trial using high-dose tamoxifen (120 mg per day) to treat HCC patients did not improve their survival length [29], questioning the relevance of ER-mediated signaling in HCC. A possible explanation for the negative result may be the lack of proper patient selection according to ER expression. Rather, tamoxifen may also act in HCC via an ER-independent pathway. On the other hand, tamoxifen could be effective only in a selected subgroup of HCC patients with the presence of variant estrogen receptors (vER-) [30]. Tamoxifen could not be effective in tumors with vER-, because of its inability to bind the receptor, and this could contribute to justify tamoxifen lack of efficacy, considering that a relevant proportion of HCC patients have predominant vERs [30]. To date, there is no robust evidence to consider tamoxifen a part of the current managements of HCC.

## 3. Progesterone and Related Compounds

### 3.1. Progesterone

Progesterone is a 21-carbon hormone formed from steroid precursors in the ovary, testes, adrenal gland, placenta, and glial cells in the central nervous system [41]. It is present in highest concentrations in the ovarian corpus luteum. In nonpregnant women, the main sites of progesterone biosynthesis are the ovaries and the adrenal cortices [42]. The synthesis of progesterone is stimulated by luteinizing hormone (LH), which primarily acts to regulate the conversion of cholesterol to pregnenolone, a progesterone precursor. 

Although the administration of progesterone to human beings gives rise to the excretion of pregnanediol in the urine, the course and sites of the metabolism of progesterone have not been established. When progesterone is administered orally, it first undergoes metabolism in the gut, then the intestinal wall, and the liver to form its hydroxylated metabolites and their sulfate and glucuronide derivatives [43, 44]. The uterus and ovaries are not essential for the reduction of progesterone, since a rise in urinary pregnanediol has been demonstrated in men and in hysterectomized women who were injected with progesterone. However, the liver would appear to be important in metabolizing the hormone. It has been shown in animals that when progesterone is implanted in the spleen, mesentery, or stomach or injected into the portal vein, its biological potency is much lower than when administered subcutaneously. The data presented indicate that progesterone is metabolized by an enzyme system in the liver tissue under the conditions used in these experiments [45]. The urinary progesterone derivatives were assumed to result from metabolism in the liver and included 5*β*-pregnanes such as pregnanediol (5*β*-pregnane-3*α*,20*α*-diol) and pregnanolone (5*β*-pregnan-3*α*-ol-20-one) as well as the 5*α*-pregnanes, 5*α*-pregnane-3,20-dione (5*α*P), 5*α*-pregnan-3*α*-ol-20-one, 5*α*-pregnan-3*β*-ol-20-one, and 5*α*-pregnan-3-3*α*(*β*), 20*α*-diols [46]. The rapid metabolism of intravenously administered [14C] progesterone by eviscerated rats [47, 48], in which tissues such as liver, spleen, gut, and adrenals had been removed, showed that progesterone conversion was also occurring extrahepatically. It then soon became apparent that progesterone serves as the precursor for the major steroid hormones (androgens, estrogens, and corticosteroids) produced by the gonadal and adrenal cortical tissues. These progesterone-metabolizing enzymes included 5*α*-reductase, 5*β*-reductase, 3*α*-hydroxysteroid oxidoreductase (3*α*-HSO), 3*β*-HSO, 20*α*-HSO, 20*β*-HSO, 6*α*(*β*)-, 11*β*-, 17-, and 21-hydroxylase, and C17–20-lyase [49]. 

The biological activity of natural progesterone and its binding of AR are controversial. There are reports showing that progesterone have relative binding activity of dihydrotestosterone (DHT), an androgen hormone, with agonist and antagonist activity [50, 51]. On the other hand, it has been reported that progesterone binds the AR with very low affinity or does not bind the AR at all, displaying no androgenic effects but weak antiandrogenic effects in animal models [52, 53]. The antiandrogenic effect is considered as a competitor in inhibition of 5*α*-reductase activity thereby decreasing the conversion of testosterone to the more active DHT but not the binding of androgen receptor [52]. However, until now, there is no clinical evidence for AR-mediated androgenic and antiandrogenic activity of progesterone [54].

### 3.2. Megestrol Acetate

Megestrol acetate (MA) is a 17*α*-acetoxy-6-dehydro-6-methylprogesterone and sometimes abbreviated as MGA or MA, which is a steroidal progestin and progesterone derivative (specifically, 17*α*-hydroxylated progesterone) with predominantly progestational and antigonadotropic effects [55]. It has been suggested that the remarkably enhanced hormonal activity of progesterone when substituted at C-6 and C-17 in the steroid nucleus is due to increased resistance to metabolizing enzymes. Preliminary experiments with MA, a potent orally active ovulation inhibitor, indicated that it was very resistant to metabolism *in vitro* by rat liver as compared with progesterone [56].

 MA acts predominantly as a potent agonist of the PR to exert its effects [57]. In addition, MA can suppress hormone-dependent tumoral cells, though the biological mechanisms underlying its antitumoral activity are not well understood. The growth-inhibitory effects on the cell cycle are not phase-specific, but its activity appears to reach a peak in the G1 phase of cell division [58]. As a potent antiestrogen agent that acts at the postreceptor level and thus independent of ER, MA is used in the second-line management of carcinoma of the breast. However, Fu et al. has revealed that the motility and invasiveness of breast cancer cells (T47D) was increased under MPA stimulation via recruiting extranuclear signaling to actin, which leads to rearrangement of the cytoskeleton and the formation of pseudopodia and membrane ruffles [37] (Table 1). It has been reported to cause minor reduction of tumor size and prolonged survival time in HCC [35] (Table 1). In experimental animal models, however, it has been shown that MA could only inhibit the growth of PR-positive tumors but not PR-negative tumors [59–61]. 

Furthermore, it produces detectable androgenic effects in animals only at a dose that is the equivalent of approximately 200 times that typically used for the treatment of prostate cancer in men [62].

### 3.3. Medroxyprogesterone Acetate

Medroxyprogesterone acetate (MPA) is a 17*α*-hydroxy-6*α*-methylprogesterone acetate, and commonly abbreviated as MPA, which is a steroidal progestin, a synthetic variant of the human hormone progesterone [55]. MPA is commonly used in contraception and hormone replacement therapy [63]. MPA is a potent full agonist of the AR. Its activation of the AR has been shown to play an important and major role in its antigonadotropic effects and in its beneficial effects against breast cancer [64–66]. In fact, likely due to its suppressive actions on androgen levels, it has been reported that MPA is highly effective in improving preexisting symptoms of hirsutism in women with the condition [67, 68]. Moreover, MPA rarely causes any androgenic effects in children with precocious puberty, even at very high doses [69]. The reason for the general lack of virilizing effects with MPA, despite its binding to and activating the AR with a high affinity and this action playing a crucial role in many of its physiological and therapeutic effects, is not entirely clear. However, MPA has been found to interact with the AR in a fundamentally different way than other agonists of the receptor such as dihydrotestosterone (DHT) [51]. The result of this difference is that MPA binds to the AR with a similar affinity and intrinsic activity to that of DHT but requires about 100-fold higher concentrations for a comparable induction of gene transcription, while at the same time not antagonizing the transcriptional activity of normal androgens like DHT at any concentration [51]. This may explain the low propensity of MPA for producing androgenic side effects. 

The intrinsic activities of MPA in activating the PR and the AR have been reported to be at least equivalent to those of progesterone and dihydrotestosterone (DHT), respectively, indicating that it is a full agonist of these receptors [51].

## 4. Progesterone Signaling

PR is a member of the nuclear receptor family of ligand-dependent transcription activators and is expressed as two different sized proteins from a single gene by alternate promoter usage. The two PR isoforms, PR-A and PR-B, are identical in their DNA binding domains (DBD) and C-terminal ligand binding domains (LBD), differing only in the N-terminal domain that is truncated in PR-A [70, 71]. Notably, PRs are found in the uterus, central nervous system, mammary gland, and pituitary gland.

The general pathway of progesterone-inducible PR-mediated gene transcription has been well characterized. Progesterone binding induces a conformational change(s) in PR that promote dissociation from a multiprotein chaperone complex, homodimerization, and binding to specific progesterone response elements (PREs) within the promoter of target genes [72, 73]. In cancer cells, kinase signaling initiated by extracellular progesterone modulates transcriptional events in the nucleus, which in turn regulate proliferation, migration, and invasion [74]. The major biological response to progesterone is mediated by PR-A and PR-B through distinct signaling pathways [75, 76]. In general, PR-B is a stronger transcriptional activator, whereas PR-A can function as a ligand-dependent repressor of other steroid hormone receptors including PR-B and ER [77]. In addition to direct transcriptional effects mediated by nuclear PR, other authors have shown that progestins can rapidly activate the Src/Ras/MAPK, PI3 kinase/Akt, and JAK2/Stat3 signaling pathway in breast cancer and mammary epithelial cells [31, 78–86] (Table 1). Many of them have been demonstrated in HCC [87]. However, their relation to progesterone signaling in HCC has not been explored so far. Progesterone also exerted a stimulatory effect through the PR on the induction of reactive oxygen species (ROS) generation processes and intracellular pathways, resulting in TGF-beta1 expression, rat hepatic stellate cells (HSCs) activation, and fibrogenic effects [88]. This may raise the possibility that progesterone could establish a tumor-favorable microenvironment and thus contributes to hepatocarcinogenesis. Further investigations are required. These effects of progestins on cell signaling pathways in the absence of transcription are dependent on conventional PR, suggesting that PR has dual functions as a nuclear transcription factor and as a modulator of cell signaling pathways. Human PR contains a polyproline SH3 domain interaction motif within the NTD in a position (aa 421–428) that is shared by PR-A and PR-B [79]. Therefore, the ability of PR to interact with Src appears to be a function of the receptor distinct from its transcriptional activity and is dissociable by point mutations in the SH3 domain interaction motif [89]. Notably, progestin activation of Src/MAPK signaling can regulate selected target genes such as cyclin D1 (CCND1) that lack direct PR binding response elements (PREs) [89]. Furthermore, progestin induction of CCND1 was observed in cells expressing PR-B but not PR-BΔSH3 or PR-A. In contrast progestin induction of Sgk (serum and glucocorticoid regulated kinase) gene, which contains a classical PRE, was observed with both PR isoforms as well as PR-BΔSH3 and was unaffected by Src and MAPK inhibitors. It is suggested the importance of PR activation of extranuclear signaling pathways in regulating selected target genes and cell cycle progression. The previous study provided evidence that c-Src is often activated in the early stage of human HCC, especially in low proliferating activity, but not in noncancerous liver tissues regardless of their histological types. More interestingly, activated c-Src was not detected in 12 atypical adenomatous hyperplasia occurring in liver cirrhosis, which has been thought to be a representative precursor for HCC [90]. 

The two putative progesterone membrane receptors PGRMC (progesterone receptor membrane component) 1 and 2 are indentified in various human tissues including liver [91]. PGRMC1 and PR are likely to be continuously active in high presence of serum progesterone [92]. Interestingly, PGRMC1 is regarded as a biomarker for tumor cell proliferation [93] and is strongly expressed in different kinds of cancer [92]. In granulosa cells, PGRMC1 mediated the antiapoptotic action of progesterone [94]. Recent publications describe an interaction of PGRMC1 with a wide range of cytochrome P450 proteins [95]. This is remarkable as PGRMC1 was proposed to be involved in chemotherapy resistance, a well-known characteristic in HCC. 

Drug-induced liver injury (DILI) is a major safety concern in drug development and clinical drug therapy. It is generally believed that women exhibit worse outcomes from DILI than men. Intriguingly, evidence showed that progesterone exacerbated the immunomediated hepatotoxic responses in DILI via the Kupffer cells and extracellular signal-regulated kinase (ERK) pathway [96]. Progesterone pretreatment dramatically activated ERK in HAL-induced liver injury, and U0126 (ERK inhibitor) significantly suppressed the exacerbating effect of progesterone and the expression of inflammatory mediators. The study seemed to provide a link between progesterone and some inflammatory mediators including tumor necrosis factor (TNF) *α*, interleukin (IL)-1*β*, and IL-6, which have been associated with HCC development.

## 5. Clinical Application of Progesterone Compounds in Hepatocellular Carcinoma

MA has powerful antiandrogenic and antiestrogenic effects in humans at sufficient doses, capable of decreasing circulating androgen and estrogen concentrations to castrate levels in both sexes and significantly lowering the expression of the AR and the ER in the body [97–99]. Rather, MA is a high-affinity, weak partial agonist/antagonist of the AR [100–102], where it binds with very similar but slightly less affinity relative to the PR [57]. At clinical doses in humans, it appears to behave purely as an antiandrogen. No androgenic side effects have been observed with the use of MA in patients of either sex at doses up to as high as 1,600 mg per day [103]. A report of a phase II study of MA (160 mg/day, orally) in the treatment of HCC showed there were no complete responders or partial responders. Twelve patients (38%) of the enrolled 56 patients had stable disease and seven of these patients had a minor response with a median size reduction in the tumor of 18%. Twenty patients (62%) had progressive disease. Five of 24 (21%) patients had a median reduction in alpha-fetoprotein levels of 59 ng/mL. The overall median survival was 4 months (range 1 week to 27 months). Twenty of 32 (62%) patients had an increased appetite and a feeling of well-being. Fourteen of 22 (64%) patients had a median lean bodyweight gain of 5 kg (range 1–14 kg) [35] (Table 1). Rather, MA was able to favorably influence such severe course, significantly improving survival, which increased from 7 to 18 months, and slowing down tumor growth in inoperable HCC [32] (Table 1). In contrast, Chow et al. indicated that MA has no role in prolonging OS (overall survival) in advanced treatment-naive HCC [36] (Table 1). 

MPA commonly is used in contraception and hormone replacement therapy [63]. Liver metastases from breast cancer are present in about 20% of patients at the time of the diagnosis of metastatic disease [104]. Faced with patients with liver metastases in whom the tumor shows positive ER and/or PR, hormonal therapy can have an important therapeutic contribution, if combined with chemotherapy and, in selected cases, even as a single therapy [104]. MPA acts as an agonist of the progesterone, androgen, and glucocorticoid receptors (PR, AR, and GR, resp.) [105]. However, few of these may include faulty patient subset selection criteria, no monitoring of tumor ER and AR expression at the time of recruitment and also during treatment of these patients and lastly the type of hormonal treatment given to the patient. Therefore, the debatable potential of hormone therapy in HCC may finally be attributed to the lack of complete understanding of ER and AR expression and hormonal responsiveness in the liver and their involvement in development of HCC [106]. Some clinical studies found that the use of MPA after hepatectomy was a strong predictor of the overall survival of patients with HCC, although the use of MPA was imperfectly associated with a better overall survival of patients with HCC [39] (Table 1). The leptin expression may intensify the curative effect of MPA in patients with HCC and may serve as a predictor for response to treatment with MPA. Nevertheless, this finding requires further investigation [39] (Table 1). Both MA and MPA are belonging to 17alpha-hydroxyprogesterone derivatives, they share a similar chemical structure and almost have the same enzymatic activity including progestogenic, antigonadotropic, antiestrogenic, androgenic, and glucocorticoid. A major difference is that MA have antiandrogenic activity but not MPA [105]. Previous study has shown that MA inhibits the growth of HepG2 cells *in vitro* in dose- and time-dependent manners. The growth of HepG2 cell-transplanted tumors in nude mice was also inhibited by i.p. injection of MA. Rather, expression of PR was not detected at protein and mRNA levels in HepG2 [33] (Table 1). MA can also exert its action on ER pathways at postreceptorial level. In HCC patients with variant ER expression, MA can temporarily suppress tumor size and increase again after three month during the follows up time [27] (Table 1). Additionally, out of 133 patients diagnosed with HCC and screened for eligibility, 45 patients (33.3%) had variant ER transcripts demonstrated in the tumor and were enrolled in the study. Twenty-four patients were randomized to no treatment and 21 to MA at the daily dose of 160 mg. Median survival in untreated patients was 7 mo (95% CI, 3.01–10.99 mo) versus 18 mo (95% CI, 13.47–22.53 mo) in patients treated with MA (*P* = 0.009) [34] (Table 1), suggesting that MA improves HCC progression may via other hormone receptors, such as androgen and glucocorticoid receptor [64, 102]. However, an animal model experiment in rat HCC showed that in the group treated with MPA no significant curative effects were observed [38] (Table 1). Tamoxifen- (TAM-) and MPA-combined chemotherapy may not prolong the survival of patients with HCC, although it improves their quality of life [40] (Table 1). Notably, AR, ER, and PR, members of steroid hormone receptor, are known to exist in human HCC [34, 38] (Table 1). 

## 6. Conclusion

Epidemiological reports have indicated that, regardless of etiologies, the incidence of HCC is higher in males than in females with the male : female ratio usually averaging between 2 : 1 and 4 : 1 [2]. It is presumably possible that sex hormones may play roles in HCC risk or development. Rather, rare information regards the potential involvement of progesterone in HCC. We introduce these studies and hope that one can notice the role of progesterone in HCC. It is noted that high levels of progesterone are observed in patients with cirrhosis, one of premalignant lesion [18] and is likely due to major metabolism of progesterone performed in the liver. In addition, progesterone can serve as the precursor for the major steroid hormones (androgens, estrogens, corticosteroids) produced by the gonadal and adrenal cortical tissues, while men have a higher incidence of HCC than women which might be resulted from the stimulatory effects of androgen and the protective effects of estrogen.

The biological activity of natural progesterone on the HCC is controversial and lacks clear investigation. The presence or absence of PR in HCC also seemed not to contribute to clinical features [24]. Rather, progesterone can rapidly activate the Src/Ras/MAPK, PI3 kinase/Akt, and JAK2/Stat3 signaling pathway in breast cancer [78–86]. Many of them have been demonstrated in HCC [87]. Intriguingly, a synthetic progestin, MA, caused minor reduction of tumor size and prolonged survival time in HCC [35] (Table 1). The growth of HepG2 cell-transplanted tumors in nude mice was also inhibited by i.p. injection of MA [33] (Table 1). A major difference is that MA have antiandrogenic activity but not MPA [105]. However, an animal model experiment in rat HCC showed that in the group treated with MPA no significant curative effects were observed [38] (Table 1). Some clinical studies found that the use of MPA after hepatectomy was imperfectly associated with a better overall survival of patients with HCC. Rather, the leptin expression may intensify the curative effect of MPA in patients with HCC and may serve as a predictor for response to treatment with MPA. Nevertheless, this finding requires further investigation [39] (Table 1).

Taken together, we believe that progesterone may have roles in HCC risk and development. Further investigations are required. In addition, monitoring of tumor PR, ER, and AR expression at the time of recruitment will be important. 

## Figures and Tables

**Table 1:** *In vitro*,* in vivo*, and clinical effects of progesterone and its related compounds in HCC.

Progestin	Bioeffect and physical response	Reference
Progesterone	Activation of Src and downstream MAPK induced Elk-1. Transactivation that was nearly as efficient as Elk-1 activation by EGF increase in the % of cells in G2M+ S phase	[31]

MA	Significant decreased tumor growth and improved survival in treated patients than the placebo group	[32]
	Inhibition of the growth of HepG2 in dose- and time-dependent manner, and HepG2 transplanted tumor *in vivo *	[33]
	HCC patients who received MA treatment would have longer median survival (18 months) compared to untreated patients (7 months)	[34]
	MA improves HCC patients' appetite, bodyweight, and a feeling of well-being with minimal side effects. And a minor reduction of tumour size and a prolonged survival	[35]
	Efficiency of MA treatment can be determined by expression of variant ER in HCC, but MA shows only a temporary inhibition of tumor growth	[27]
	MA has no role in prolonging OS in advanced treatment-naive HCC	[36]

MPA	Increased migration and invasion	[37]
	No significant curative effects were observed in MPA-treated HCC rat	[38]
	Expression level of leptin predicts postoperative treatment efficiency of MPA in HCC patients	[39]
	Tamoxifen- and MPA-combined chemotherapy may not prolong the survival of patients with HCC, although it improves their quality of life	[40]
